# Prenylated Flavonoids from *Morus alba* L. Cause Inhibition of G1/S Transition in THP-1 Human Leukemia Cells and Prevent the Lipopolysaccharide-Induced Inflammatory Response

**DOI:** 10.1155/2013/350519

**Published:** 2013-05-20

**Authors:** Peter Kollar, Tomáš Bárta, Jan Hošek, Karel Souček, Veronika Müller Závalová, Shushan Artinian, Rabih Talhouk, Karel Šmejkal, Pavel Suchý, Aleš Hampl

**Affiliations:** ^1^Department of Human Pharmacology and Toxicology, Faculty of Pharmacy, University of Veterinary and Pharmaceutical Sciences Brno, Palackého 1-3, 612 42 Brno, Czech Republic; ^2^Department of Histology and Embryology, Faculty of Medicine, Masaryk University, Kamenice 3, 625 00 Brno, Czech Republic; ^3^Department of Natural Drugs, Faculty of Pharmacy, University of Veterinary and Pharmaceutical Sciences Brno, Palackého 1-3, 612 42 Brno, Czech Republic; ^4^Department of Cytokinetics, Institute of Biophysics, Academy of Sciences of the Czech Republic, v.v.i., Královopolská 135, 612 65 Brno, Czech Republic; ^5^International Clinical Research Center, St. Anne's University Hospital, Pekařská 53, 656 91 Brno, Czech Republic; ^6^Nature Conservation Center, American University of Beirut, P.O. Box 11-0236, Beirut 1107 2020, Lebanon; ^7^Department of Biology, Faculty of Arts and Sciences, American University of Beirut, P.O. Box 11-0236, Beirut 1107 2020, Lebanon

## Abstract

*Morus alba* L. (MA) is a natural source of many compounds with different biological effects. It has been described to possess anti-inflammatory, antioxidant, and hepatoprotective activities. The aim of this study was to evaluate cytotoxicity of three flavonoids isolated from MA (kuwanon E, cudraflavone B, and 4′-*O*-methylkuwanon E) and to determine their effects on proliferation of THP-1 cells, and on cell cycle progression of cancer cells. Anti-inflammatory effects were also determined for all three given flavonoids. Methods used in the study included quantification of cells by hemocytometer and WST-1 assays, flow cytometry, western blotting, ELISA, and zymography. From the three compounds tested, cudraflavone B showed the strongest effects on cell cycle progression and viability of tumor and/or immortalized cells and also on inflammatory response of macrophage-like cells. Kuwanon E and 4′-*O*-methylkuwanon E exerted more sophisticated rather than direct toxic effect on used cell types. Our data indicate that mechanisms different from stress-related or apoptotic signaling pathways are involved in the action of these compounds. Although further studies are required to precisely define the mechanisms of MA flavonoid action in human cancer and macrophage-like cells, here we demonstrate their effects combining antiproliferative and anti-inflammatory activities, respectively.

## 1. Introduction

The root bark of *Morus alba* L. (Moraceae; white mulberry) is used for its diuretic, antitussive, antidiabetic, and antipyretic effects in world traditional medicine [1]. Therefore, *Morus* species plants have been intensively studied from phytochemical point of view, and bioactive compounds of flavonoid character have been isolated. Within the flavonoid class of natural products, the prenylated subclass is quite rich in structural variety and pharmacological activities. Compounds obtained from *M. alba* L. possess anti-inflammatory, antibacterial, antiviral, antioxidant, and hepatoprotective activities [2–6]. Extracts obtained from *M. alba* L. were evaluated for their cytotoxicity against various tumor cells, such as K-562, B380 human leukemia cells, and B16 mouse melanoma cells [7]. Several studies have been published in which bioactive compounds isolated from white mulberry exerted potent effect on human cancer cell lines. Morusin, one of the most efficient substances, showed strong activity against cervical carcinoma HeLa, breast carcinoma MCF-7, and hepatocarcinoma Hep3B cells [8]. Anticancer mechanism of morusin action in colorectal HT-29 cells is proposed to be mediated by induction of apoptosis and by suppression of NF-*κ*B activity [9]. Another mulberry constituent, albanol A, induces apoptotic cell death in HL60 leukemia cell line via both the cell death receptor pathway by stimulation of death receptor and the mitochondrial pathway by topoisomerase II inhibition through caspase 2 activation [10].

The connection between inflammation and cancer can be thought of as consisting of two pathways: an extrinsic mechanism, where a constant inflammatory state contributes to increased cancer risk (such as in an inflammatory bowel disease), and an intrinsic mechanism, where acquired genetic alterations (such as activation of oncogenes) trigger tumor development [11]. The NF-*κ*B signaling plays crucial roles in both precancerous chronic inflammation as well as cancer induced inflammation. An activation of this pathway induces expression of inflammatory cytokines, adhesion molecules, enzymes involved in the prostaglandin-synthesis pathway (such as COX-2), inducible nitric oxide synthase (iNOS), angiogenic factors, and antiapoptotic genes (such as Bcl-2) [12]. Proinflammatory cytokines implicated in carcinogenesis include, for instance, IL-1, IL-6, IL-15, colony stimulating factors (CSF), or TNF-*α* [13].

**Figure 1:**
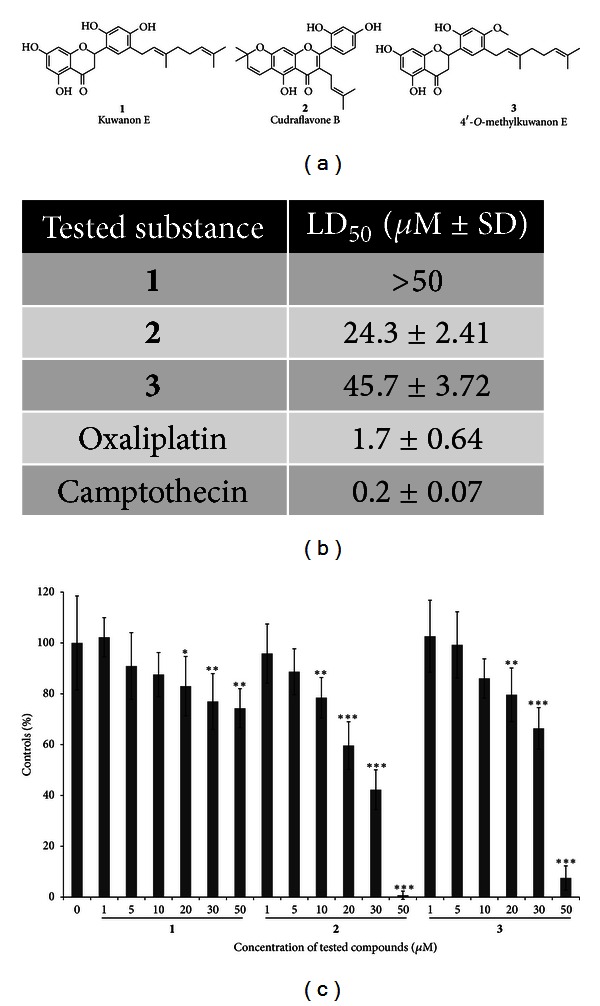
Toxicity and inhibitory effects of tested MA compounds on THP-1 leukemia cell proliferation. (a) Structure of *M. alba* L. prenylated flavonoids. (b) THP-1 cells were seeded (2 × 10^5^ cells/mL), treated with the indicated concentrations of **1**, **2**, and **3** for 24 h, cell numbers were counted, and viability was determined by erythrosin B exclusion. Toxicity was expressed as the LD_50_ values. (c) THP-1 cells were seeded (5 × 10^4^ cells/well) in 96-well plates. Proliferation of cells was determined using WST-1 assays. Bars represent the proliferation of cells cultured in the presence of increasing concentrations of MA compounds as a percentage of controls at 24 h. The results shown are expressed as the means ± S.D. of three independent experiments, with each condition tested in triplicate (*P < 0.05, **P < 0.01, and ***P < 0.001).

We have previously identified prenylated and geranylated flavanone compounds from plants of Moraceae and Paulowniaceae families with cytostatic activity in normal human fibroblasts and five human cancer cell lines [14]. Furthermore, we clarified the underlying molecular mechanisms mediating the effects of geranylated flavanone tomentodiplacone B on cell growth [15]. We showed that tomentodiplacone B induced accumulation of human monocytic leukaemia (THP-1) cells in G1 phase of cell cycle, which was in concert with downregulation of the cyclin E1 isoform and cyclin A2 levels, reduced CDK2 activity, and reduced pRb phosphorylation [15]. Our most recent work focusing on prenyl flavonoid cudraflavone B, which is contained in large amounts in the roots of white mulberry, showed unusually pronounced anti-inflammatory properties of this compound [2]. Moreover, throughout the course of experiments, we found that cudraflavone B had a strong effect on proliferation of human macrophage-like cells. It was therefore interesting to evaluate its effect on cell cycle progression and to elucidate the mechanisms of its cell proliferation inhibitory action. However, besides cudraflavone B (given designation** 2**) we also isolated and characterized two other prenylated flavanones from *M. alba* L., which we have identified as kuwanon E (**1**), and 4′-*O*-methylkuwanon E (**3**), a new compound detected and described in our laboratory. Structures of all three tested *M. alba* L. prenylated (geranylated) flavonoids are shown in Figure 1(a). Based on our preliminary pilot data and the literature search (structure-effect relationship) we expected cytotoxic effect via targeting the cell cycle kinetics and viability.

The aim of our work was to evaluate effect of prenylated and geranylated flavonoids isolated from *M. alba* L. on proliferation of THP-1 cells and also to determine cell cycle profiles in several human cancer cells treated with *M. alba* L. flavonoids. As the role of inflammation in cancer is recently intensively discussed, we have also assessed anti-inflammatory effects of the previously mentioned flavonoids.

## 2. Methods

### 2.1. Test Compounds and Reagents

All three tested compounds (**1**, **2**, and **3**) were isolated and supplied by the Department of Natural Drugs, Faculty of Pharmacy, University of Veterinary and Pharmaceutical Sciences Brno, Czech Republic. The identification of substances was carried out using HRMS, ^1^H, and ^13^C NMR analyses,and their purity exceeded 95% according to the HPLC analysis [2, 14]. These compounds are poorly soluble in water; therefore, fresh 10 mM stock solutions in dimethylsulfoxide (DMSO) (Sigma-Aldrich, St. Louis, MO, USA) were prepared 1 day prior to experiments and stored at −20°C. These solutions were further diluted in the culture media to the desired final concentrations. RPMI 1640, DMEM, and IMDM culture media, phosphate buffered saline (PBS), and antibiotics (penicillin and streptomycin) were purchased from Lonza (Verviers, Belgium). Foetal bovine serum (FBS), phorbol myristate acetate (PMA), prednisone (purity ≥ 98%), and the lipopolysaccharide (LPS) obtained from *Escherichia coli *0111:B4 were purchased from Sigma-Aldrich. Instant ELISA Kits (eBioscience, Vienna, Austria) were used to evaluate the production of TNF*α* and IL-1*β*. Cytoscreen Kit (BioSource Europe S.A., Nivelles, Belgium) was used to detect IL-1RA cytokine by ELISA method. Mouse monoclonal antibody against cyclin B1 (MS-868) was purchased from Neomarkers (Fremont, CA, USA). Mouse monoclonal antibody against cyclin A (sc-53228) was purchased from Santa Cruz Biotechnology (Santa Cruz, CA, USA). Rabbit polyclonal antibodies against poly(adenosine diphosphate (ADP) ribose) polymerase (PARP), caspase 3, and phospho histone H3 were purchased from Cell Signaling Technologies (Beverly, MA, USA). Mouse monoclonal antibody against *γ*-H2AX [pS139] (05-636) was purchased from Millipore (Billerica, MA, USA). Mouse monoclonal antibodies against pRb (554136) were purchased from BD Biosciences (Franklin Lakes, NJ, USA). Rabbit polyclonal antibody against pRb [pT821] (44-582G) was purchased from BioSource (Carlsbad, CA, USA). Parthenolide (PTL), oxaliplatin, cisplatin and camptothecin, and all other reagents were purchased from Sigma-Aldrich.

### 2.2. Cell Culture

The human monocytic leukemia THP-1 cell line was purchased from the European Collection of Cell Cultures (Salisbury, UK; methods of characterization: DNA fingerprinting (multilocus probes) and isoenzyme analysis). Cells were cultured in RPMI 1640 medium supplemented with antibiotics (100 U/mL penicillin, 100 mg/mL streptomycin), 10% FBS, and 2 mM L-glutamine. Cultures were kept in an incubator at 37°C in a water-saturated 5% CO_2_ atmosphere in air. Cells were passaged at approximately 1-week intervals. Cells were routinely tested for the absence of mycoplasma infection (Hoechst 33258 staining method). Mouse mammary epithelial cell line, SCp2 cells (kindly provided by P. Y. Desprez, Geraldine Brush Cancer Research Institute, California Pacific Medical Center, San Francisco, CA, USA), was cultured in DMEM supplemented with insulin 5 *μ*g/mL (Sigma, St. Louis), 1% penicillin/streptomycin mixture (Lonza Walkersville, Inc., USA), and 5% heat inactivated FBS (Sigma-Aldrich), in a humidified incubator (95% air, 5% CO_2_) at 37°C [16]. THP-1 cells were split into 24-well plates to achieve concentration of 100 000 cells/mL and were differentiated to macrophages by a phorbol myristate acetate (PMA) as described previously [17]. PC3 and DU-145 cells were obtained from the American Type Culture Collection (ATCC). PC3 and DU-145 were cultured in RPMI-1640, Ham's F12, or McCoy's media, respectively (Gibco Invitrogen Corporation, Carlsbad, CA, USA) with 2 mM L-glutamine, streptomycin (0.1 mg/mL), and penicillin (100 U/mL), and supplemented with 10% fetal bovine serum. LAPC-4 cells [18], a generous gift of Dr. R. Reiter (UCLA, Los Angeles, CA, USA), were cultured in Iscove's Modified Dulbecco's Medium (IMDM, Invitrogen) supplemented with NaHCO_3_, penicillin/streptomycin, 10% FBS, and 1 nM R1881 (PerkinElmer). Benign prostatic hyperplasia (BPH) epithelial cells BPH-1 [19] were obtained from the German Collection of Microorganisms and Cell Cultures. The cells are androgen unresponsive and were cultured in RPMI 1640 (Invitrogen), supplemented with 20% bovine fetal serum (PAA Laboratories, Pasching, Austria), 5 *μ*g/mL transferrin, 5 ng/mL sodium selenite, 5 *μ*g/mL insulin (Invitrogen), streptomycin (0.1 mg/mL), and penicillin (100 U/mL) (PAA). Cells were cultured at 37°C in a humidified 5% CO_2_ incubator.

### 2.3. *In Vitro* Analysis of Cytotoxicity and Cell Proliferation

THP-1 cells were seeded (2 × 10^5^ cells/mL) and incubated for 24 h at 37°C with 5% CO_2_ with tested compounds dissolved in DMSO (Sigma-Aldrich) in concentrations ranging from 1 to 50 *μ*M in RPMI 1640 medium. The maximum concentration of DMSO in the assays never exceeded 0.1%. Numbers and viabilities of the cells were determined by counting with a hemocytometer as we previously described [15]. Cell proliferation was determined using a WST-1 assay kit (Roche Diagnostics, Mannheim, Germany) according to the manufacturer's instructions. For WST-1 assays, cells were seeded into 96-well plates (5 × 10^4^ cells/well in 100 *μ*L culture medium) in triplicates in complete RPMI 1640 medium, and measurements were taken 24 h after adding the tested MA compounds. All data were evaluated using GraphPad Prism 5.00 software (GraphPad Software, San Diego, CA, USA, http://www.graphpad.com/).

### 2.4. Cell Cycle Analysis

Cancer THP-1, LAPC-4, DU-145, PC3 cells, and human nontumorigenic benign prostatic hyperplasia BPH-1 cells were incubated with increasing concentrations of tested MA compounds for 24 h, washed in PBS (pH 7.4), and fixed for 30 min in an ice-cold 70% ethanol. Fixed cells were washed three times in PBS (pH 7.4) and incubated with RNase A (0.02 mg/mL) (Boehringer, Ingelheim, Germany) for 30 min at 37°C. Nuclei were stained with propidium iodide (40 *μ*g/mL) and analysed by flow cytometry using a Beckman Coulter Cytomics FC500 flow cytometer (Beckman Coulter, Brea, CA, USA). Cell cycle distribution was analysed using FlowJo software (http://www.flowjo.com/).

### 2.5. Western Blotting

Cells were washed three times with PBS (pH 7.4) and lysed in 100 mM Tris-HCl (pH 6.8) containing 20% glycerol and 1% SDS. Protein concentrations were determined using the DC Protein Assay Kit (Bio-Rad, Hercules, CA, USA). Lysates were supplemented with bromophenol blue (0.01%) and *β*-mercaptoethanol (1%). Equal amounts of total protein were separated by SDS-polyacrylamide gel electrophoresis (PAGE), electrotransferred onto PVDF membranes (Millipore, Billerica, MA, USA), immunodetected using the appropriate primary and secondary antibodies, and visualised with ECL Plus reagent (Amersham, Aylesbury, UK) according to the manufacturer's instructions.

### 2.6. Treatment of THP-1 Cells with Drugs, Induction of Inflammatory Response, and Determination of Cytokines Production

Macrophages differentiated from THP-1 cells were pretreated for 1 h with tested compounds dissolved in DMSO to obtain final concentrations of 10 *μ*M (this concentration lacked cytotoxic effect). For comparison with conventional drugs, 1 *μ*M prednisone dissolved in DMSO was used. Vehicle-treated cells contained a vehicle (DMSO) only. The concentration of DMSO was 0.1% in each well. The inflammatory response was triggered by adding LPS dissolved in water (1 *μ*g/mL) to drug-pretreated macrophages, and cells were incubated for another 24 h. After this time period, medium was collected and the concentration of cytokines was measured by ELISA assay. The lowest detection limit was 7.8 pg/mL for TNF-*α* and 31.3 pg/mL for both IL-1*β* and IL-1RA. LPS-untreated cell served as controls.

### 2.7. Treatment of SCp2 Cells with Drugs, Induction of Inflammatory Response, and Zymography

SCp2 cells were plated in a 24-well plate in density of 4 × 10^4^ cells/mL. After 24 h of incubation in medium containing 5% FBS, the medium was replaced, the cells were washed by PBS and fresh media supplemented with 1% FBS, and tested compounds were added. Final concentrations of tested compounds were 5 *μ*M (this concentration lacked cytotoxic effect (data not shown)). Vehicle-treated and control cells were prepared using the same protocol as for THP-1 macrophages. For comparison with conventional drugs, parthenolide (5 *μ*M dissolved in DMSO) was used, because of its usual use as a control for this type of cells and experiments and its well-known ability to inhibit the expression of matrix metalloprotease (MMP)-9 [20]. The inflammatory response was triggered by adding a nontoxic dose of LPS (10 *μ*g/mL) to the drug-pretreated SCp2 cells, which were then incubated at 37°C for another 24 h [21]. After this time period, medium was collected and the pro-MMP-2 and MMP-2 activity was measured by zymography as described previously by Talhouk et al. [22]. Briefly, 20 *μ*L of collected medium was loaded into polyacrylamide gel impregnated by 0.1% gelatin. After electrophoresis, SDS from gels was washed out by 2.5% Triton X-100, and gels were incubated for 30 min at room temperature (~23°C) and overnight (16–20 h) at 37°C in developing buffer (50 mM Tris (pH 8.8), 5 mM CaCl_2_, 3 mM NaN_3_, and 0.5% Triton X-100). Gels were then stained by Coomassie blue [22]. Intensity of digested regions was determined by densitometry followed by calculation using AlphaEaseFC 4.0.0 software (Alpha Innotech, USA). It should be noted that the conditioned medium contained active MMP-2, which represented 75.3% of measured activity. Therefore, this value was subtracted from all obtained results of MMP-2 activity.

### 2.8. Statistical Analysis

Statistical significance was tested using the one-way ANOVA with Dunnett's test and Tukey post test for comparisons between the means, and differences between two conditions were retained for *P* < 0.05. Statistical significance was determined at levels of *P* < 0.05, *P* < 0.01, and *P* < 0.001.

## 3. Results

### 3.1. Cytotoxic and Growth Inhibitory Effects of **1**, **2**, and **3** on THP-1 Cells

To determine the effects of all three tested substances obtained from *M. alba* on the viability and growth of human leukemia cells, the THP-1 cells were exposed for 24 h to increasing concentrations (1, 2.5, 5, 10, 20, and 50 *μ*M) of **1**, **2**, and **3**, respectively, stained for viability, and counted by hemocytometer. From this data, the LD_50_ values for each MA compound were calculated (Figure 1(b)). Toxicity expressed as LD_50_ increased as follows: **1** (>50 *μ*M), **3** (45.7 ± 3.72), and **2** (24.3 ± 2.41). To compare toxicity of MA compounds with already known chemical or natural substances, we assessed LD_50_ of oxaliplatin (1.7 ± 0.64) and camptothecin (0.2 ± 0.07), and, in both, it showed much lower toxic concentration values. Subsequent WST-1 assay, determining cell number using metabolic activity as a readout following exposure to MA compounds for 24 h, revealed that proliferation of THP-1 cells was inhibited by all three tested substances. As shown in Figure 1(c), substance **2** exhibited the strongest effect, as 10 *μ*M and higher doses caused dose-dependent inhibition of THP-1 cell growth. The significant reduction of metabolic activity (*P* < 0.05) was though observed in cells treated with each of the three flavonoids at concentrations of 20 *μ*M or higher. Based on cytotoxicity and proliferation data, the concentration range of MA compounds from 5 to 30 *μ*M was selected for all subsequent experiments.

### 3.2. Effects of **1**,  **2**, and **3** on Distribution of Cells in Cell Cycle Phases

**Figure 2:**
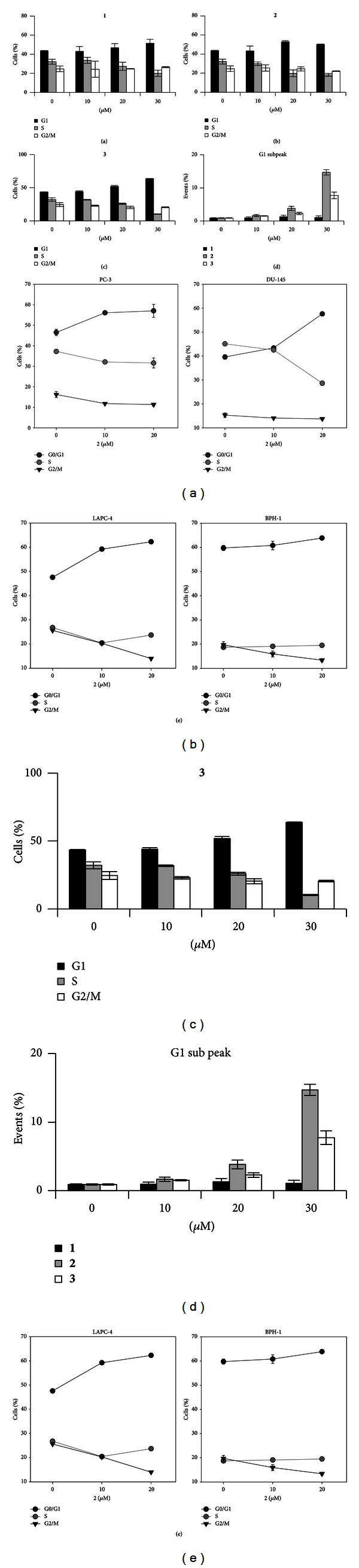
Treatment with *M. alba *L. prenylated flavonoids causes accumulation of several cancer cells in G1 phase. (a) Cell cycle distribution at 24 h upon treatment of THP-1 cells with **1** as determined by flow cytometry. (b) Cell cycle distribution at 24 h upon treatment of THP-1 cells with **2** as determined by flow cytometry. (c) Cell cycle distribution at 24 h upon treatment of THP-1 cells with **3** as determined by flow cytometry. (d) Quantification of G1 subpeak in MA flavonoids treated THP-1 cells. (e) Cell cycle distribution at 24 h upon treatment of cancer PC3, DU-145, LAPC-4, and immortalized BPH-1 cells with **2** as determined by flow cytometry.

In order to investigate the effect of tested substances on the cell cycle progression, we performed cell cycle analysis based on DNA content using flow cytometry of THP-1 cells. The data shown in Figures 2(a)–2(c) demonstrate that all compounds tested (**1**, **2**, and **3**) accumulate human leukemia cells in G1 phase dose-dependently after 24 h treatment. While the percentage of S phase cells decreased, the percentage of cells with 4N DNA content, representing G2/M phase, was unchanged upon treatment with tested compounds. This effect was dominant in substance **3**, lasting even after 72 h (data not shown).

Since compound **2** exerted the strongest impact on viability and proliferation, together with impact on the cell cycle profile of THP-1 cells (observed already from 10 *μ*M concentration), we have expanded our analysis with this substance to further 3 human cancer cell lines (Figure 2(e)). The inhibition of G1/S transition, accompanied by the decreased proliferation caused by **2**, was observed in all cancer cell lines used in this experiment (LAPC-4—metastatic prostate, established from lymph nodes in SCID mice; PC3—androgen receptor null, p53-null, metastatic (bone) prostate cancer; and DU145—androgen receptor, p53-mutated, metastatic (brain) prostate cancer). To assess whether **2** affects also the cell cycle of human nontumorigenic cell line, we exposed the prostate epithelial BPH-1 cells, derived from the benign prostatic hyperplasia, to this compound. Interestingly, the distribution of BPH-1 cells in all three cell cycle phases remained unchanged even after the treatment with high concentrations of **2** used in the study (20 and 30 *μ*M) (Figure 2(e)).

Although a G1 subpeak in a DNA histogram detected by flow cytometry cannot be considered as specific hallmark of apoptosis, it represents besides cellular debris also the apoptotic population of cells [23, 24]. The appearance of the G1 subpeak was increased at 24 h after beginning the treatment with MA compounds, although with a different intensity of this effect (Figure 2(d)). While **1** exerted no G1 subpeak increase, the strongest induction of apoptosis was found in 30 *μ*M **2**-treated THP-1 cells (~15-fold higher compared to control). Significant increase of G1 subpeak (~8-fold higher compared to control) was caused also by 30 *μ*M **3** compound. Nevertheless, even the highest concentration of **2** used did not cause such massive apoptosis that we found in 5 or 10 *μ*g/mL cisplatin, included as a model compound (~36- and ~60-fold higher, resp., compared to control).

### 3.3. Expression of Cell Cycle Regulators in MA-Treated Cells

**Figure 3:**
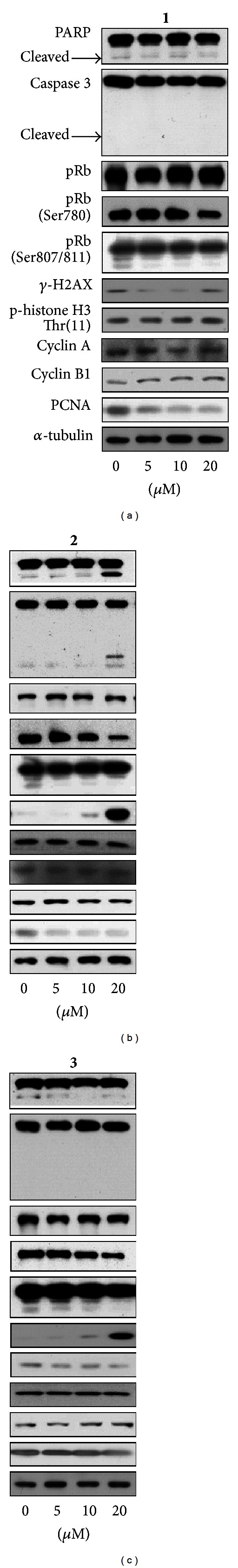
Expression of cell cycle regulators and stress response proteins after 24 h of (a) **1**, (b) **2**, and (c) **3** treatment.

Based on the fact that all tested compounds cause accumulation of cells in G1 phase, we determined the expression and phosphorylation status of key cell cycle and stress-related proteins. Phosphorylated Rb protein is the key regulatory molecule, which coordinates processes critical for G1/S progression. We therefore examined whether pRb phosphorylation is suppressed in THP-1 cells treated by MA compounds. As shown in Figures 3(a) and 3(c), 24 h exposure to 20 *μ*M **1** or **3** results in reduced phosphorylation of Rb protein on serine 780. For **2** this effect was even more pronounced (Figure 3(b)). Phosphorylation on serines 807/811 was also decreased in THP-1 cells exposed to MA compounds, in clearly dose-dependent manner (Figures 3(a)–3(c)). It is highly probable that MA-induced Rb dephosphorylation corresponds to the accumulation of cells in G1 phase.

Another protein involved in cell cycle machinery, which we analyzed in MA-treated cells, was proliferating cell nuclear antigen (PCNA). This protein is well known as a DNA sliding clamp for DNA polymerase delta and as an essential component for eukaryotic chromosomal DNA replication and repair [25]. All flavonoids tested downregulated the expression of PCNA in THP-1 cells (Figures 3(a)–3(c)), again correspondingly to the observed decrease of cells in S phase of the cell cycle.

Cyclins A and B are members of the cyclin family, expression of which fluctuates during cell cycle progression peaking in S and G2 phases, respectively. We found that none of the tested MA compounds affects the quantity of these cyclins, when measured in asynchronously growing cells. Moreover, phosphorylation of histone H3 at threonine 11, which normally peaks at M phase, remained unaffected even after 24 h treatment with MA compounds. Unchanged phosphorylation of histone H3 with normal expression of cyclins A and B suggests that MA compounds do not influence progression through G2 and M phases of cell cycle.

Caspase 3-mediated PARP cleavage has been considered as a hallmark of apoptosis. It is also known that PARP activation is induced by DNA strand breaks [26]. Neither **1** nor **3** did cause PARP cleavage, and so its activation in THP-1 cells. However, increased histone *γ*-H2AX phosphorylation together with cleavage of both caspase 3 and PARP in **2**-treated THP-1 cells indicates the activation of the stress signaling apoptotic pathways caused by the highest concentration used (20 *μ*M).

### 3.4. Behaviour of Inflammatory Response Markers in MA-Treated Cells

**Figure 4:**
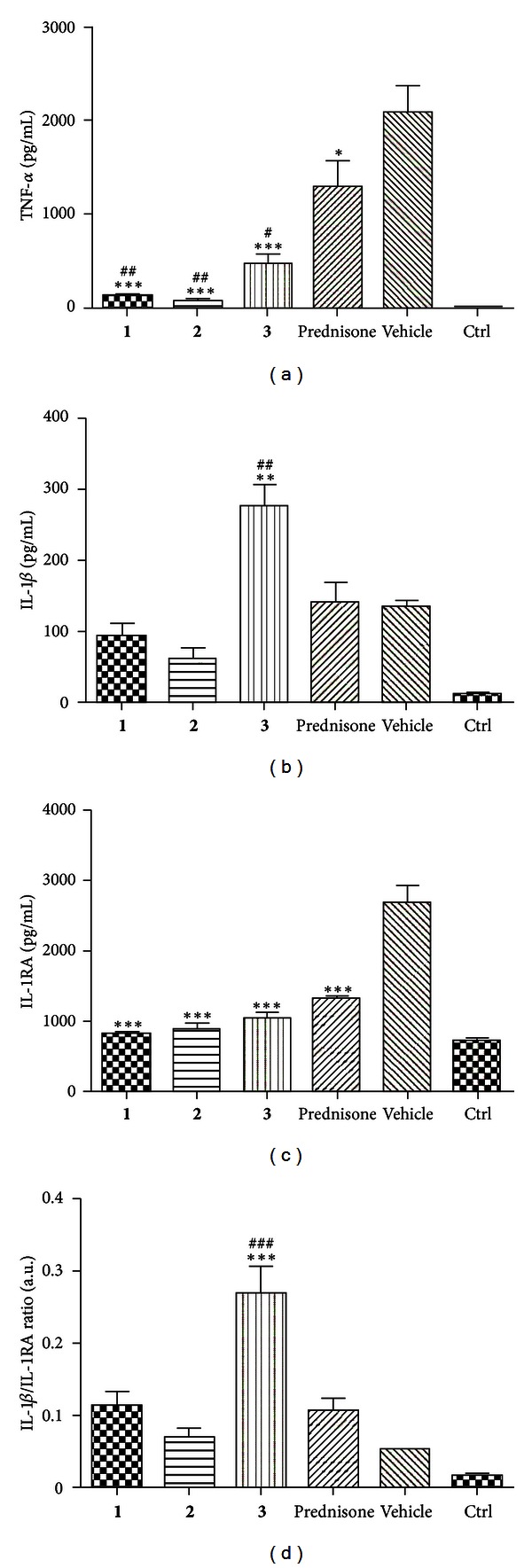
(a) Effects of *M. alba *L. prenylated flavonoids and the reference drug prednisone on LPS-induced TNF-*α* secretion at macrophages derived from THP-1 cell line. Cells were pretreated with given compounds (10 *μ*M), prednisone (1 *μ*M), or the vehicle (DMSO) only. After 1 h of incubation, the inflammatory response was induced by LPS (except for the control cells). Results are expressed as means ± S.E. for three independent experiments. *Significant difference in comparison to vehicle-treated cells (*P* < 0.05), ***significant difference in comparison to vehicle-treated cells (*P* < 0.001), ^#^significant difference in comparison to prednisone-treated cells (*P* < 0.05), and ^##^significant difference in comparison to prednisone-treated cells (*P* < 0.01). (b) Effects of *M. alba *L. prenylated flavonoids and the reference drug prednisone on LPS-induced IL-1*β* secretion at macrophages derived from THP-1 cell line. Cells were pretreated with given compounds (10 *μ*M), prednisone (1 *μ*M), or the vehicle (DMSO) only. After 1 h of incubation, the inflammatory response was induced by LPS (except for the control cells). Results are expressed as means ± S.E. for three independent experiments. **Significant difference in comparison to vehicle-treated cells (*P* < 0.01); ^##^significant difference in comparison to prednisone-treated cells (*P* < 0.01). (c) Effects of *M. alba *L. prenylated flavonoids and the reference drug prednisone on LPS-induced IL-1RA secretion at macrophages derived from THP-1 cell line. Cells were pretreated with given compounds (10 *μ*M), prednisone (1 *μ*M), or the vehicle (DMSO) only. After 1 h of incubation, the inflammatory response was induced by LPS (except for the control cells). Results are expressed as means ± S.E. for three independent experiments. ***Significant difference in comparison to vehicle-treated cells (*P* < 0.001). (d) Ratio IL-1*β*/IL-1RA production calculated for macrophages derived from THP-1 cell line. Values were obtained from ELISA measurements of individual cytokines as it is described in Figures 2 and 3. Results are expressed as means ± S.E. for three independent experiments. A.U. = arbitrary unit. ***Significant difference in comparison to vehicle-treated cells (*P* < 0.001); ^###^significant difference in comparison to prednisone-treated cells (*P* < 0.001).

**Figure 5:**
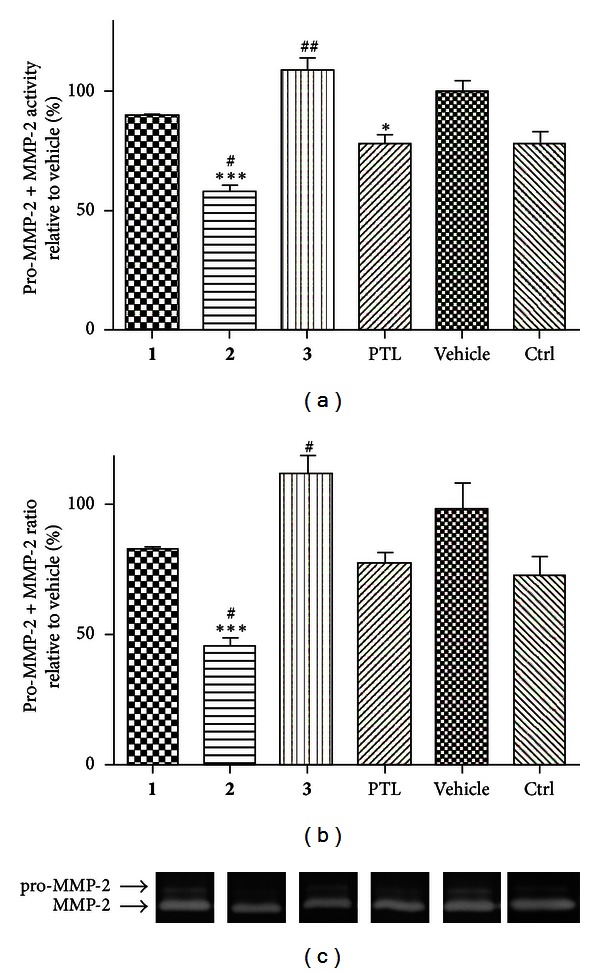
Effects of *M. alba *L. prenylated flavonoids and the reference drug parthenolide (PTL) on LPS-induced (pro-)MMP-2 activity at SCp2 cells. Cells were pretreated with given compounds (5 *μ*M), parthenolide (5 *μ*M), or the vehicle (DMSO) only. After 1 h of incubation, the inflammatory response was induced by LPS (except for the control cells). Activity of (pro-)MMP-2 was detected by zymography (a). Intensity of digested bands was analyzed by densitometry. (b) shows pro-MMP-2/MMP-2 ratio. Showed gels represent results of three independent experiments (c). Results are expressed as means ± S.E. for three independent experiments. ***Significant difference in comparison to vehicle-treated cells (*P* < 0.001), ^#^significant difference in comparison to parthenolide-treated cells (*P* < 0.05), and ^##^significant difference in comparison to parthenolide-treated cells (*P* < 0.01).

Protein TNF-*α* together with other cytokines, such as interleukins, not only plays crucial role in the inflammatory response but also is involved in carcinogenesis [13]. To investigate whether antiproliferative effects of MA compounds are accompanied by anti-inflammatory activity, we assessed levels of selected inflammatory response markers secreted into the culture medium by LPS-activated macrophages derived from THP-1 cell line. As evident from Figure 4(a), LPS-induced TNF-*α* secretion by macrophages was reduced upon the treatment with MA compounds, similarly to prednisone used as the reference anti-inflammatory drug. Notably, all three compounds tested were significantly more effective than prednisone. Levels of IL-1*β*, the most studied member of the IL-1 family [27], produced by THP-1-derived macrophages were slightly decreased by tested substances, except for **3** (Figure 4(b)). Treatment with this compound (10 *μ*M) led to significant (*P* < 0.01) increase of IL-1*β* secreted to cell culture medium. The natural antagonist of IL-1*β* is IL-1RA, and their mutual ratio is crucial for a progression of inflammation and maintaining a homeostasis. All tested flavonoids, similarly to prednisone, significantly decreased the secretion of IL-1RA (Figure 4(c)). This secretion attenuation affected the IL-1*β*/IL-1RA ratio (Figure 4(d)). This increase was nonsignificant for compounds **1**, **2**, and prednisone. On the other hand, 4′-*O*-methylkuwanon E (**3**) increased the IL-1*β*/IL-1RA ratio by the factor of 5.33. It is caused by enormously elevated secretion of IL-1*β*. Matrix metalloproteinase 2 (MMP2) is involved in the tissue development and remodelling, but it also contributes to inflammation progression. It is secreted as inactive pro-MMP2 form, which is extracellularly cleaved to its active form. The amount of (pro-)MMP2 was significantly decreased only by **2** and the control drug parthenolide (PTL) (Figure 5(a)) in SCp2 cell line. Whereas PTL inhibits proteinase activity to the level typical for unstimulated cells, **2** was able to reduce the (pro-)MMP2 activity below these control cells. **2** uniquely and significantly decreased the pro-MMP2/MMP2 ratio (Figure 5(b)).

## 4. Discussion and Conclusions

Relevance of the crosstalk between components of the immune system and cancer cells is widely discussed. During the last decade the clear evidence that inflammation plays a critical role in tumorigenesis has been obtained, and some of underlying molecular mechanisms have been elucidated [28]. A role of inflammation in tumorigenesis is now generally accepted, and it has become evident that an inflammatory microenvironment is an essential component of all tumors, including some in which a direct causal relationship with inflammation is not yet proven [11].

In the present study, we assessed cytotoxicity and the effects of three prenylated (geranylated) flavonoids from *M. alba* L., kuwanon E (**1**), cudraflavone B (**2**), and 4′-*O*-methylkuwanon E (**3**) on cell cycle progression and selected cell cycle regulatory proteins. We have also extended our study with the aim of evaluating the effect of these substances on proinflammatory markers, because we recently reported that **2** has potent anti-inflammatory properties in human macrophages [2]. Compounds are poorly soluble in water; therefore, we used DMSO as a solvent. The final DMSO concentration of 0.5–1% is frequently employed in *in vitro* studies to solubilize/deliver bioactive compounds to cells. However, it has been shown that DMSO exhibits a myriad of biological actions, such as reported effects on cell cycle, differentiation, inflammatory response, and apoptosis studies [29–31]. Since our intention was focused on evaluation of these types of effects, it was necessary to take into account the effects of DMSO in arrangement of all conducted experiments. In particular, the concentration of DMSO in experiments never exceeded 0.1%. Moreover, to minimalize misinterpretations of our results due to biological effects of DMSO, we employed DMSO-only-treated THP-1 cells as controls in each experiment setting. Based on our previously published results [14, 15, 17] we used human monocytic leukaemia cells THP-1 as a model system to detect cytotoxic and cytostatic effects of newly isolated natural compounds and THP-1-derived macrophages for studies on inflammatory response. We found strong antiproliferative effects of all three tested MA compounds in concentrations ranging from 10 to 50 *μ*M. When comparing these data with the LD_50_ values, we may conclude that unlike **2**, both **1** and **3** at concentrations of 20 *μ*M and 30 *μ*M had significant growth inhibitory effect without being cytotoxic to the cells. As regards substance **2**, we speculate that the observed reduction of metabolic activity is more likely a sign of cell dying rather than growth inhibition.

To reveal whether antiproliferative effects seen in THP-1 cells after 24 h treatment with MA flavonoids reflect inhibition of cell transition between specific cycle phases, we conducted the cell cycle analysis. Our results showed that all tested compounds caused accumulation of THP-1 human leukemia cells in G1 phase of cell cycle (and inhibited their entry into the S phase) in a dose-dependent manner. Taking into account the strength of **2** effect on viability, proliferation, and the cell cycle profile (showed from the concentration of 10 *μ*M, in contrast to other MA substances), we exposed three other human cancer as well as nontumorigenic cell lines to **2**. While in all tumor cells **2** exhibited inhibitory effect on the G1/S transition, in nontumor line (prostate epithelial BPH-1 cells) such activity was not observed. This might indicate a partially selective effect of this substance on tumor versus nontumor cells. Nevertheless, such selectivity of **2** would have to be verified by more detailed analysis.

The cell cycle analysis allowed us to study the percentage of THP-1 cells in specific phase, including determining sub-G1 peak, which covers also cells undergoing the process of apoptosis. One of the characteristic events of apoptosis is the proteolytic cleavage of poly(ADP-ribose)polymerase (PARP), a nuclear enzyme involved in DNA repair, DNA stability, and transcriptional regulation. Caspases, in particular caspases 3 and 7, cleave the 116-kDa form of PARP at the DEVD site to generate an 85- and a 24-kDa fragment [26]. PARP is inactivated by caspase 3 cleavage (in a specific domain of the enzyme) during programmed cell death. One-day treatment with **1** had no effect on induction of apoptosis as determined by flow cytometry assessment of G1 subpeak and western blot analysis of PARP and caspase 3 cleavage. Significant increase of G1 subpeak (~8-fold higher compared to control) was caused by 30 *μ*M **3** compound; however, no cleavage of PARP and caspase 3 was observed (Figure 3(c)). Conversely, massive increase in G1 subpeak (~15-fold higher compared to control), together with occurrence of both apoptotic markers (cleaved PARP and subsequently caspase 3), was observed in cells exposed to **2** for 24 h. However, effects of **2** on THP-1 cells are not comparable with those of cisplatin, added as a model anticancer drug. Cisplatin caused considerably more substantial changes in both G1 subpeak accumulation and caspase 3 cleavage (see Figure S1 in Supplementary Material Available online at http://dx.doi.org/10.1155/2013/350519), suggesting that **2** mechanism of action is not similar to that of platinum derivatives. These results prompted us to experimentally address molecular mechanisms underlying the effects of MA compounds on cell growth.

Cyclins A and B are members of the cyclin family, with the maximum of their expression during S and G2 phases of a cell cycle. Cyclin A is required for cell to progress through the S phase, and cyclin B is necessary for cells to enter mitosis and divide into two daughter cells [32]. It is also known that activation of tumor suppressor retinoblastoma protein (pRb) permits transcription of key S-phase-promoting genes, including some that are required for DNA replication. In contrast, dephosphorylation of pRb slows the progression of cells into S phase [33]. None of the MA compounds tested were found to reduce, after 24 h exposure, the expression of cyclins A and B. This fact, together with the reduced pRb phosphorylation caused by all MA compounds, possibly indicates that these substances affect rather the G1/S than G2/M transition. Flow cytometry data further support this hypothesis, since significant accumulation of cells in G1 at the expense of S phase was observed upon the treatment with MA compounds. Since such cell cycle distortion could be mediated by stress response signaling pathways, their activation was evaluated in THP-1 cells treated by all three MA flavonoids. In THP-1 cells stress-associated regulators such p21, p27, and p53 proteins are not detectable [15]. Therefore, we focused on histones *γ*-H2AX (becomes phosphorylated on damaged DNA) and H3 (its phosphorylation on Thr11 correlates with mitotic/meiotic chromosome condensation). Cells treated with any MA compound displayed no changes in phosphorylation of histone H3 on the given residue. For *γ*H2AX we observed increased phosphorylation only in cells treated with **2** at concentration of 20 *μ*M, which is the same as such causing cleavage of caspase 3 and PARP. Collectively, we speculate that **2** exerts mode of action that is different from that of **1** and **3**. Compound **2** seems to inhibit proliferation via triggering the stress-related pathway leading to Rb dephosphorylation and apoptosis with typical cleavage of PARP and caspase 3. On the other hand, no induction of the stress-related proteins occurs in **1**- and **3**-treated cells, and **1** in all tested concentrations clearly affects PCNA, which facilitates and controls DNA replication, and is at the very heart of cell-cycle progression.

As mentioned at the beginning, our previous study on effects of cudraflavone B (**2**) in human macrophages showed an interesting anti-inflammatory activity of this flavonoid. Since newly characterized compounds **1** and **3** were also isolated from *M. alba* L., and chemically belong to the same category, we expected similar effects. Yet, except secretion of TNF-*α* and IL-1RA, we found different results after application of MA substances to macrophages. Therefore, only relatively little structural differences between compounds tested (presence of 2,3 double bond at **2**, presence and position of prenyl or geranyl side chains, or substitution of flavonoid *B*-ring) strongly affect the mechanism of action and play a role in final effect of compound.

Importantly, our results pointed to huge differences among the tested compounds. **1** and **2** showed similar inhibition effect on TNF-*α*, IL-1*β*, and IL-1RA expression. On the other hand, **3**, which differs from **1** by substitution of one hydroxyl group on the *C*-ring for methoxy group, attenuated only TNF-*α* and IL-1RA expression, but less effectively than **1** or **2**, and secretion of IL-1*β* was strongly elevated. It is obvious that all three compounds are able to downregulate expression of genes that are under transcriptional control of NF-*κ*B. In comparison with other two cytokines, IL-1*β* is synthetized as proprotein, and it is cleaved into active form by caspase-1-containing inflammasome [34]. Increased IL-1*β* production in the presence of LPS in cells was observed following incubation with doxorubicin and daunorubicin [35] or Cu(II) complexes [36]. We cannot exclude the possibility that **3** activates an inflammasome, and, thus, augments IL-1*β* secretion. It should be noted that although **1** and **2** inhibited IL-1*β* secretion, the effect is much smaller than in the case of TNF-*α*. This may indicate that all three compounds are able to positively regulate inflammasome action. The low ability of tested compounds to downregulate proinflammatory IL-1*β* and significant downregulation of anti-inflammatory IL-1RA are showed in higher IL-1*β*/IL-1RA ratio. The MMP-2 activity is in agreement with TNF-*α* and IL-1*β* expression—**2** significantly decreased its level, **1** inhibited its activity only slightly, and **3** moderately raised its level. According to these results, the highest antiphlogistic potential has **2** followed by **1**. Depending on conditions, flavonoids can act both as prooxidants and antioxidants. The ability to cause dysfunction of mitochondria by prooxidant effect is connected with possible mechanisms of anticancer action, which may lead to apoptosis of tumor cells. Their antioxidant activity is connected with direct scavenging effect of excessive reactive oxygen/nitrogen species or with interaction with enzymes involved in their production or elimination. Interaction with enzymes responsible for carcinogen activation can lead to prevention of tumor formation [37]. Only a few reports on anti/prooxidative activity of compounds analysed in this study have been published. In general, these compounds do not fulfil the structural requirements needed for direct scavenging effect *in vitro* [38], which was confirmed for compound **2** [6, 39]. Park et al. [40] showed only weak activity of **2** in protecting LDL particles against oxidation (TBARS assay), but the inhibition of NO formation mediated via inhibition of iNOS was proved using RAW 264.7 cells. Protective effects of prenylated compounds (**1** and **3**) against oxidative stress-induced damage of human neuroblastoma SH-SY5Y cells were observed, showing their potential antioxidant activity [41]. Compound **1** showed inhibitory activity on NO production in RAW 264.7 cells [42]. Possible pro/antioxidant activity of tested compounds and its interconnection with their anticancer effects should be clarified in further experiments.

In conclusion, the reported active agents isolated from *M. alba* L. have an interesting impact on human cells, which are involved in both tumor and inflammation. Of the three compounds tested, **2** showed the strongest effects on cell cycle progression and viability of tumor cells and on inflammatory response of macrophage-like cells. In addition, substances **1** and **3** exerted more sophisticated rather than direct toxic effect on used cell types. Our data indicate that mechanisms different from stress-related or apoptotic signaling pathways are involved in the action of these compounds. Although further studies are required to precisely define the mechanisms of MA flavonoid actions, here we clearly demonstrate their effects combing antiproliferative and anti-inflammatory activities in human cancer and macrophage-like cells, respectively. Confirmed dual activity of tested prenylated flavonoids could be an inspiration for chemical modifications of their structures or isolation of similar substances in order to get more potent agents usable for clinical practice in future.

## Supplementary Material

Cisplatin, strong inducer of apoptosis, is commonly used chemotherapy drug. In our experimental settings, we included cisplatin as a model compound to compare its effects on apoptosis with those of MA flavonoids. As shown in Supplementary Figure 1, 24 h treatment with cisplatin caused in THP-1 cells significant changes in G1 sub-peak accumulation and in both apoptotic markers (cleaved PARP and caspase 3).

## Abbreviations

MA: *Morus alba* L.
**1**: Kuwanon E
**2**: Cudraflavone B
**3**: 4′-*O*-methylkuwanon E
PBS: Phosphate buffered saline
FBS: Fetal bovine serum
DMSO: Dimethylsulfoxide
PMA: Phorbol myristate acetate
LPS: Lipopolysaccharide
PTL: Parthenolide.
