# Supplementary figures and images for: Prenylated Flavonoids from Morus alba L. Cause Inhibition of G1/S Transition in THP-1 Human Leukemia Cells and Prevent the Lipopolysaccharide-Induced Inflammatory Response
Source: Evid Based Complement Alternat Med. 2013 May 20;2013:350519. doi: 10.1155/2013/350519 (PMC3671669; doi:10.1155/2013/350519)

Supplementary Fig 1A-B


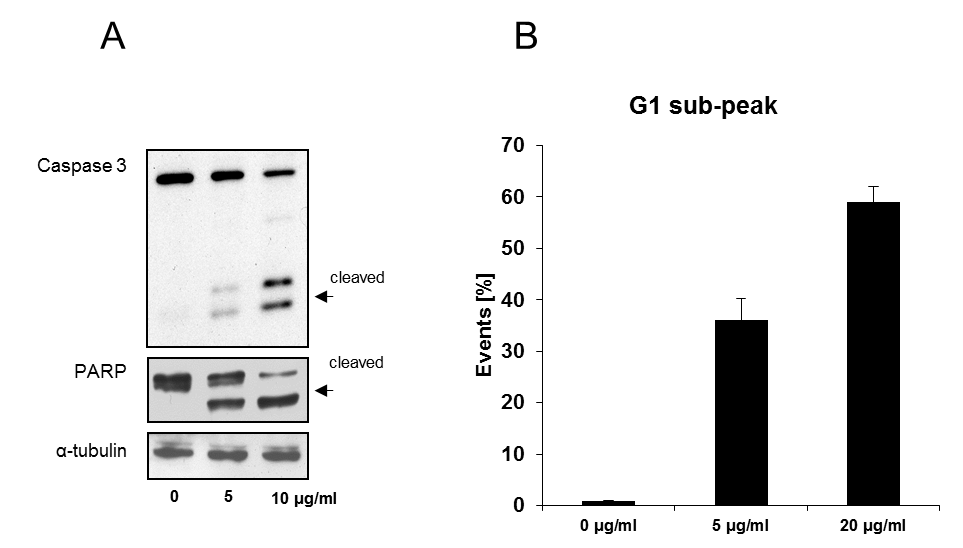

Supplement: Supplementary file 1 — Cisplatin, strong inducer of apoptosis, is commonly used chemotherapy drug. In our experimental settings, we included cisplatin as a model compound to compare its effects on apoptosis with those of MA flavonoids. As shown in Supplementary Figure 1, 24 h treatment with cisplatin caused in THP-1 cells significant changes in G1 sub-peak accumulation and in both apoptotic markers (cleaved PARP and caspase 3). [file 350519.f1.docx]
